# *Cis*-Regulation of the *CFTR* Gene in Pancreatic Cells

**DOI:** 10.3390/ijms26083788

**Published:** 2025-04-17

**Authors:** Clara Blotas, Anaïs Le Nabec, Mégane Collobert, Mattijs Bulcaen, Marianne S. Carlon, Claude Férec, Stéphanie Moisan

**Affiliations:** 1 Univ Brest, Inserm, EFS, UMR 1078, GGB, F 29200 Brest, France; megane_collobert@hotmail.fr (M.C.); claude.ferec@univ-brest.fr (C.F.); 2 School of Life Sciences, Ecole Polytechnique Fédérale de Lausanne, 1015 Lausanne, Switzerland; anais.lenabec@epfl.ch; 3 Department of Pharmaceutical and Pharmacological Sciences, KU Leuven, 3000 Leuven, Belgium; bulcaen2@gmail.com; 4 Department of Chronic Diseases and Metabolism, KU Leuven, 3000 Leuven, Belgium; marianne.carlon@kuleuven.be; 5 Leuven Viral Vector Core, KU Leuven, 3000 Leuven, Belgium; 6 Centre Hospitalier Universitaire Brest, F 29200 Brest, France

**Keywords:** CFTR, *cis*-regulatory element (CRE), 3D genome, chromatin, pancreas, tissue specificity, 4C, ATAC, H3K27ac, CRISPR/Cas9

## Abstract

Genome organization is essential for precise spatial and temporal gene expression and relies on interactions between promoters and distal *cis*-regulatory elements (CREs), which constitute ~8% of the human genome. For the *cystic fibrosis transmembrane conductance regulator* (*CFTR*) gene, tissue-specific expression, especially in the pancreas, remains poorly understood. Unraveling its regulation could clarify the clinical heterogeneity observed in cystic fibrosis and CFTR-related disorders. To understand the role of 3D chromatin architecture in establishing tissue-specific expression of the *CFTR* gene, we mapped chromatin interactions and epigenomic regulation in Capan-1 pancreatic cells. Candidate CREs are validated by luciferase reporter assay and CRISPR knock-out. We identified active CREs not only around the *CFTR* gene but also outside the topologically associating domain (TAD). We demonstrate the involvement of multiple CREs upstream and downstream of the *CFTR* gene and reveal a cooperative effect of the −44 kb, −35 kb, +15.6 kb, and +37.7 kb regions, which share common predicted transcription factor (TF) motifs. We also extend our analysis to compare 3D chromatin conformation in intestinal and pancreatic cells, providing valuable insights into the tissue specificity of CREs in regulating *CFTR* gene expression.

## 1. Introduction

Over the last decades, functional genomics has become increasingly important, particularly with the development of new technologies. They allow insights about the unexplored part of the genome, i.e., the non-coding genome [1]. The ENCODE (Encyclopedia of DNA Elements) project, launched in 2003, aims to provide a comprehensive annotation of the entire genome. The consortium estimated that 8% of the genome consists of candidate *cis*-regulatory elements, known as cCREs [2]. CREs are regions bound by transcription factors (TFs) that drive the cell type-specific expression of target genes regardless of their genomic distance. They can be classified as enhancers, silencers, insulators, or promoters, active or poised. CREs are characterized by biochemical marks such as open chromatin, nucleosome-free regions, and post-translational modifications (PTMs), such as acetylated histone H3 Lys27 (H3K27ac) for active enhancers, trimethylated histone H3 Lys4 (H3K4me3) for promoters, and CCCTC-binding factor (CTCF) binding for insulators [3]. To be functional, the regulatory region must interact with its target gene.

*Cis*-regulation is a highly conserved mechanism that is essential for correct spatio-temporal gene expression [4]. Tissue and temporal specificity make *cis*-regulation highly dynamic and challenging to study. Further research is, however, needed, both to improve knowledge and to apply these new insights to human diseases. While most disease-causing variants were previously identified in protein-coding regions, increasing evidence from whole-genome studies indicates that non-coding variants contribute to pathogenesis or confer risk in specific tissues [5,6]. It is estimated that over 90% of disease-associated variants are located in the non-coding region of the genome and may have an impact on CRE [7].

CFTR-associated diseases include cystic fibrosis (CF) and CFTR-related diseases (CFTR-RD) [8]. These conditions result from variations in the *CFTR* gene, which lead to aberrant expression or dysfunction of the encoded protein. The *CFTR* gene encodes CFTR, an ion channel responsible for conductance of chloride and bicarbonate that is expressed at the apical membrane of epithelial cell layers in several organs. Its absence or dysfunction causes a loss of ion homeostasis at the epithelial cell surface, resulting in mucus disturbance, organ obstruction, and chronic bacterial infection. Without appropriate care, these conditions can lead to death during infancy.

Significant genotypic and phenotypic heterogeneity is observed in CFTR-associated diseases, with over 2100 variants and a spectrum of clinical presentations ranging from mild to severe. CF affects multiple organs, including the lungs, small intestine, pancreas, reproductive tract, and liver. In contrast, CFTR-RD are clinical entities with features of CF and evidence of CFTR dysfunction but do not meet the criteria for a CF diagnosis [9]. The majority of these diseases manifest as mono-organ forms, including CBAVD (congenital bilateral absence of vas deferens), pancreatitis, and bronchiectasis [10]. The complexity of the clinical picture cannot be fully explained by variants in the coding sequence alone, and the regulation of the *CFTR* gene remains incompletely understood. Insight into the *cis*-regulation of the *CFTR* gene may explain at least part of this phenotypic variability.

The *CFTR* gene is surrounded by two CTCF TAD boundaries located at −80.1 kilobases (kb) upstream of the transcription start site (TSS) and +48.9 kb downstream of the last *CFTR* codon [11,12]. The first description of *CFTR* regulatory elements occurred in 1996 with the identification of a DNase I hypersensitive site (DHS) bound by TFs with enhancer activity within intron 1 of the *CFTR* gene [13]. Subsequent studies have focused on the identification of pulmonary and intestinal CREs on the basis of the severity of symptoms in these tissues and the availability of models. A comprehensive review of previous studies is provided in [14]. With respect to the pancreas, although there is a lack of knowledge and relevant models, more studies are needed, as pancreatic insufficiency represents a major manifestation in 85% of people with CF. Additionally, pancreatitis is the second most common CFTR-related disorder (CFTR-RD).

Therefore, we combine chromatin study techniques, including 4C-seq, ATAC-seq (assay for transposase-accessible chromatin), and CUT&RUN-seq (cleavage under targets and release using nuclease), to identify putative CREs in the pancreas, where little information is currently available, as recommended by Gasperini et al. [3]. The activity of the regulatory regions was assessed via a luciferase reporter assay, and we have started validating our target regulatory regions through CRISPR knock-out experiments. We identified regulatory regions previously shown to be involved in other tissues, as well as newly described regions, both within and outside the TAD. Overall, this work provides a more comprehensive regulatory landscape of the *CFTR* gene in the pancreas and highlights the tissue-specific regulation of this gene.

## 2. Results

### 2.1. Prediction Model Identifying Putative Regulatory Elements

**Figure 1:**
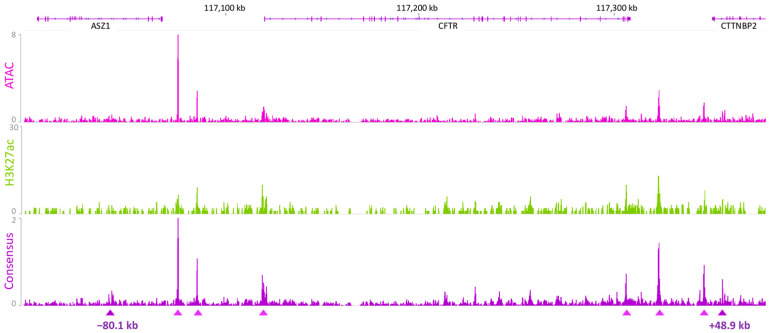
Open chromatin and enhancer mapping in Capan-1 cells. ATAC-seq allows the mapping of open chromatin regions, i.e., regions accessible to the transcriptional machinery. We applied ATAC to Capan-1 cells and identified several peaks across the locus. A peak at the *CFTR* promoter was identified, indicating the accessibility of the region and confirming that *CFTR* is expressed in Capan-1 cells. H3K27ac is an epigenetic mark for active enhancer regions. Both marks allow cell-specific identification of the active enhancer region. A consensus analysis highlighted six major peaks (purple triangles) as candidate regulatory regions in Capan-1 cells in addition to the TAD boundaries (dark purple triangles). Data were aligned to the hg19 genome.

To identify which CRE is involved in the *cis*-regulation of the *CFTR* gene in the pancreas, we performed ATAC-seq and CUT&RUN-seq for H3K27ac in Capan-1 cells. Capan-1 cells are pancreatic duct epithelial cells derived from pancreatic adenocarcinoma that express the *CFTR* gene (Figure S1) [15]. ATAC-seq allows the mapping of open chromatin regions across the entire genome. H3K27ac marks are indicative of enhancer activity. We focused our interest on the *CFTR* TAD (chr7:117,039,878–117,356,812), which was separated by the CTCF boundaries at −80.1 kb and +48.9 kb, and identified multiple peaks [11,12]. The active state of the *CFTR* promoter in Capan-1 cells was confirmed by the identification of ATAC and H3K27ac peaks in this region. A consensus analysis of the CUT&RUN and ATAC experiments resulted in a list of highly reliable peaks. In addition to the boundaries, six peaks were identified within the TAD. Two were located upstream of the promoter, one corresponded to the *CFTR* promoter, and three were located at the 3′ end of the *CFTR* gene (Figure 1).

ATAC and H3K27ac data enable the identification of active enhancer regions but do not provide information about the link between CREs and their target genes. To this end, we use the predictive activity-by-contact (ABC) model developed by Fulco et al. on the basis of a combination of experiments representing enhancer activity (ATAC-seq and H3K27ac IP) and enhancer—promoter contact frequency (Hi-C) [16,17].

**Figure 2:**
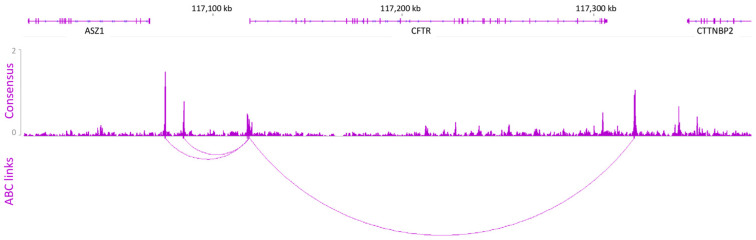
Gene–enhancer prediction links. ABC is a predictive model for identifying gene–enhancer links via open chromatin, enhancer activity, and contact frequency data. Five ABC links were identified, one of which is the promoter. Four cCREs are predicted to be involved in the regulation of the *CFTR* gene. Data were aligned to the hg19 genome.

After analysis, five ABC links were highlighted, including one involving the promoter (Figure 2). Three CREs were identified upstream of the promoter, two of which were known open chromatin regions (OCR), at −44 kb and −35 kb, and one at −114 bp of the TSS [18]. The downstream ABC link was mapped to the +15.6 kb OCR, previously described as an enhancer-blocking element [19,20].

### 2.2. Chromatin Organization Confirms the Presence of Active Regulatory Regions

**Figure 3:**
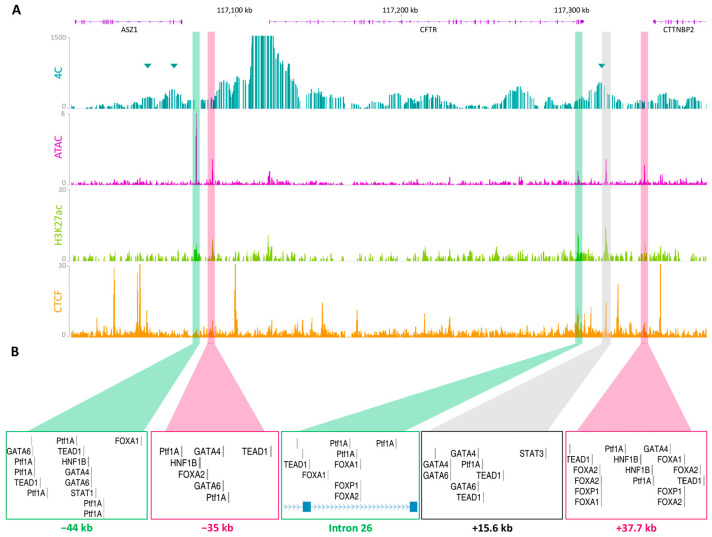
Characterization of the *CFTR cis*-regulatory landscape. (**A**) *CFTR* promoter interaction frequencies were determined via 4C-seq in Capan-1 cells. PeakC analyses highlighted significant interactions with the promoter (alphaFDR; 0.1). They are represented by blue triangles at the 5′ TAD boundaries −80.1 kb, at −44 kb, and at +15.6 kb. ATAC-seq and CUT&RUN-seq were performed for the histone marks H3K27ac in Capan-1 cells and CTCF in Caco-2 cells. All these data were integrated to map five cCREs in detail. (**B**) Pancreas-specific TFs from the Jaspar 2024 TFBS were mapped to each of them [23]. Data were aligned to the hg19 genome.

To validate regulatory interactions, 4C-seq was applied to Capan-1 cells to determine 3D chromatin contact at the *CFTR* locus. 4C-seq, developed by the de Laat group, quantifies the frequencies of chromatin interactions with a bait of interest; for this study, the *CFTR* promoter was used (Figure 3A) [21,22]. To highlight the most frequent interactions, peak calling analysis was performed on peakC (Table S1).

Among the regions identified as significant within the TAD, three were of particular interest: the −80.1 kb boundary, a region at approximately −60 kb, and the +15.6 kb region (Figure 3A, blue triangles). The interaction with the +15.6 kb region was confirmed to be highly important, in alignment with the predictions of the ABC model. However, the −44 kb and −35 kb regions identified in the ABC model do not overlap with a significant 4C peak. Nevertheless, there is a significant interaction between the promoter and upstream region, which can bring the −44 kb and −35 kb regions in proximity to the promoter. An OCR within the last intron, intron 26, of the *CFTR* gene corresponded to an H3K27ac peak as well as a 4C peak. The same observations were made for a region at +37.7 kb, which is near a previous OCR identified in the lung, the +36.6 kb region [18,24]. On the basis of this information, five cCREs were delineated. Following transcription factor (TF) alignment, putative exocrine pancreas-specific TF binding sites were confirmed in each of the five cCREs (Figure 3B).

### 2.3. Luciferase Assays Characterize cCRE Activity

**Figure 4:**
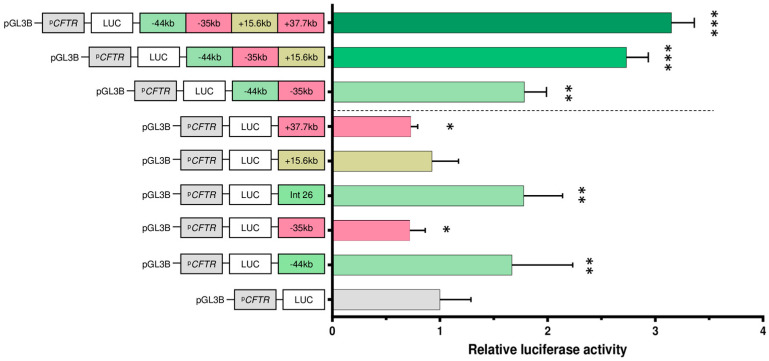
Activity assay of identified pancreatic-specific cCREs. Capan-1 cells were transfected with luciferase reporter constructs containing the *CFTR* basal promoter (PCFTR; 787 bp) and cCREs identified via chromatin analysis. Two regions, −44 kb and OCR in intron 26, show increased activity (green), whereas −35 kb and +37.7 kb show slight decreases in activity (pink). +15.6 kb shows no effect. The luciferase data are shown relative to the *CFTR* basal promoter vector (set to 1). The error bars represent the standard deviation (SD; *n* = 9), * <0.05; ** <0.001; *** <1 × 10^−9^ using unpaired *t*-tests.

To evaluate the activity and function of the candidate regulatory regions involved in *CFTR* regulation, in vitro luciferase assays were conducted. The initial analysis focused on the cCREs identified by the chromatin assay and the ABC model. The regions of interest were cloned and inserted into the pGL3-Basic vector, in which the minimal *CFTR* promoter (787 bp, chr7:117,119,328–117,120,114, hg19) drives the expression of the luciferase gene (LUC). Each construct was co-transfected into Capan-1 cells with a pCMV-beta-galactosidase control plasmid. Luciferase activity slightly increased in the presence of the −44 kb region (1.7-fold) and the OCR in intron 26 (1.8-fold) (Figure 4). Compared with the promoter alone, +15.6 kb did not affect the luciferase activity. The −35 kb and +37.7 kb regions had a minor silencing effect (−1.39-fold).

While single-CRE luciferase reporter data provide valuable information, it is important to consider the fact that in the genome, there are simultaneous promoter–CRE links; this is not an individual relationship. Several studies have shown that gene expression can be affected by one or multiple CREs [25,26,27]. Indeed, *cis*-regulation is a process whereby multiple CREs are involved in the formation of chromatin modules that in turn regulate the same gene [28]. To evaluate the cooperative effect of the identified cCREs that share putative TF binding sites, we designed three synthetic constructs: one encompassing the −44 kb and −35 kb regions; another containing the −44 kb, −35 kb, and +15.6 kb regions; and the last one with the four cCREs, −44 kb, −35 kb, +15.6 kb, and +37.7 kb. The combination of two cCREs was observed to induce an increase in luciferase activity, although this increase was not significantly different from the effect observed with the −44 kb region alone (Figure 4). When the +15.6 kb region is added, a 2.7-fold increase is measured, and with the combination of the four regions, we observe a 3.2-fold increase. The combination of CREs enhances promoter activity beyond their individual effects, suggesting a cooperative interaction between them.

**Figure 5:**
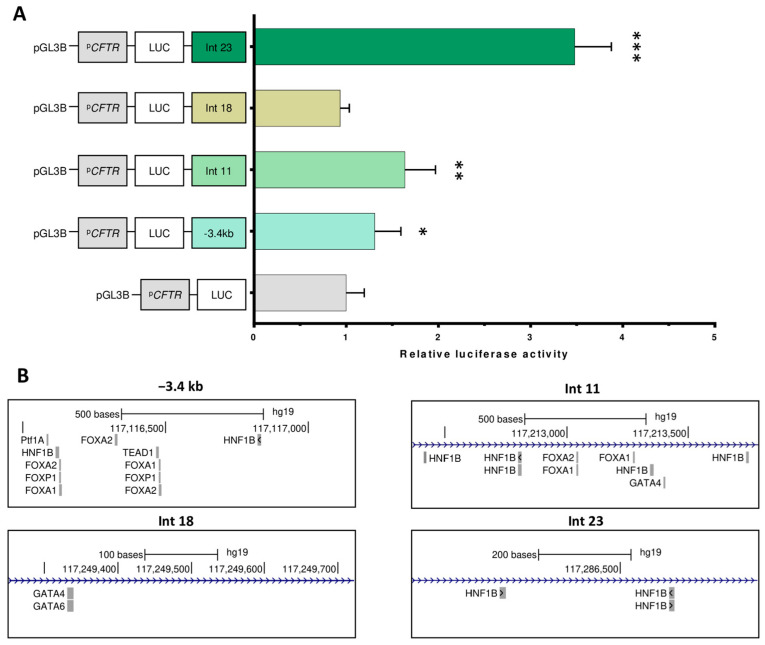
Activity assay on previously described cCREs. (**A**) Capan-1 cells were transfected with luciferase reporter constructs containing the *CFTR* basal promoter (PCFTR; 787 bp) and cCREs described in the literature. We confirmed the enhancer effect of regions −3.4 kb, the OCR in intron 11, and intron 23, with a major effect on the OCR in intron 23. The luciferase data are shown relative to the *CFTR* basal promoter vector (set to 1). The error bars represent the standard deviation (SD; *n* = 9), * 0.05; ** <0.0001; *** <1 × 10^−8^ using unpaired *t*-tests. (**B**) Pancreatic-specific TFs from the Jaspar 2024 TFBS were mapped.

To extend the functional analysis of the regulatory elements in pancreatic cells, we also tested regions that have been described as cCREs in previous studies [12,29]. Four regions were analyzed: −3.4 kb and OCRs in introns 11, 18, and 23. The −3.4 kb region has a moderate enhancing effect on pulmonary cells [30]; intron 11 is described as an enhancer in intestinal cells, and chromatin interactions have been shown in pancreatic cell lines [12,31]. OCRs have been identified in introns 18 and 23, and specific TF binding has been shown in intron 18 [29]. In our study, the OCR in intron 18 had no effect on the luciferase assay (Figure 5A). The −3.4 kb region and intron 11 seem to have a small positive effect, whereas intron 23 shows significant enhancer activity (3.5-fold). Alignment with specific exocrine pancreas TFs revealed the presence of an HNF1B motif in all cCREs, except for intron 18, which showed no effect in the luciferase assay (Figure 5B).

Finally, five active regulatory regions have been described: the region at −44 kb, at −3.4 kb, and the OCR within introns 11, 23, and 26. Two regions have minor silencing effects when present alone: the region at −35 kb and the region at +37.7 kb. Cooperative effects have been observed with the combination of CREs, with an important increase in activity.

### 2.4. Endogenous Assay Validated the Enhancer Effect of the −44 kb Region

**Figure 6:**
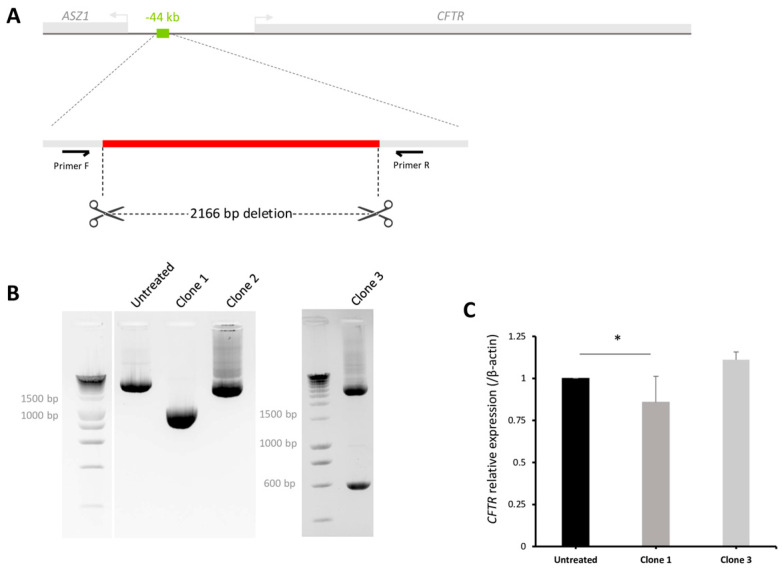
Endogenous effects of CRE deletion. (**A**) The −44 kb regulatory region was deleted by positioning two sgRNAs at each extremity. After RT-PCR and Sanger sequencing, we validated a homozygous deletion of 2166 bp shown in (**B**) clone 1 and a heterozygous deletion in clone 3. Results of RT-PCR with primers positioned outside the deleted region. Amplifications were performed on untreated cells and on the selected clone. PCR products from untreated cells are approximately 3000 bp (3239 bp). PCR products from clone 1 are approximately 900 bp, consistent with Sanger sequencing results showing a deletion of 2166 bp. Clone 2 is negative and shows the same size product as the control condition. The heterozygous deletion of clone 3 presents a WT band at 3000 bp and at 600 bp for the 2646 bp deletion. (**C**) RT-qPCR was performed on clone 1, revealing a slight decrease (15%) in *CFTR* gene expression normalized to the *β-actin* gene compared to the untreated condition. Results are based on two technical replicates and three biological replicates. * <0.05 using unpaired *t*-tests.

To confirm our observations based on chromatin structural assays (ATAC-seq, CUT&RUN-seq and 4C) and experimental quantification using the reporter assay, we performed precise deletion of the endogenous −44 kb enhancer region using CRISPR/Cas9 in Capan1 cells. Two flanking single guide RNAs (sgRNAs) were designed and positioned at each extremity of the targeted region. To achieve homozygous deletion, efficient delivery of the genome editing technology is essential. For that reason, virus-like particles (VLPs) were produced containing the −44 kb flanking sgRNAs (Figure 6A). Homozygous deletion was confirmed by PCR followed by Sanger sequencing, validating a clone with a 2166 bp deletion (Figure 6A,B).

The impact of the CRE deletion on *CFTR* gene expression was assessed by RT-qPCR and compared to untreated Capan-1 control cells. The homozygous deletion (clone 1) of the −44 kb region reduced *CFTR* expression by 15% compared to untreated cells, whereas the heterozygous deletion had no effect (Figure 6C). These findings are consistent with the results of the luciferase reporter assay, which demonstrated a slight increase in activity in the presence of the −44 kb CRE. (Figure 4). These results demonstrate that the combination of ATAC-seq, CUT&RUN-seq, 4C, and the luciferase reporter assay allows us to identify and validate new enhancer regions.

### 2.5. Exploring the Landscape Outside the TAD

**Figure 7:**
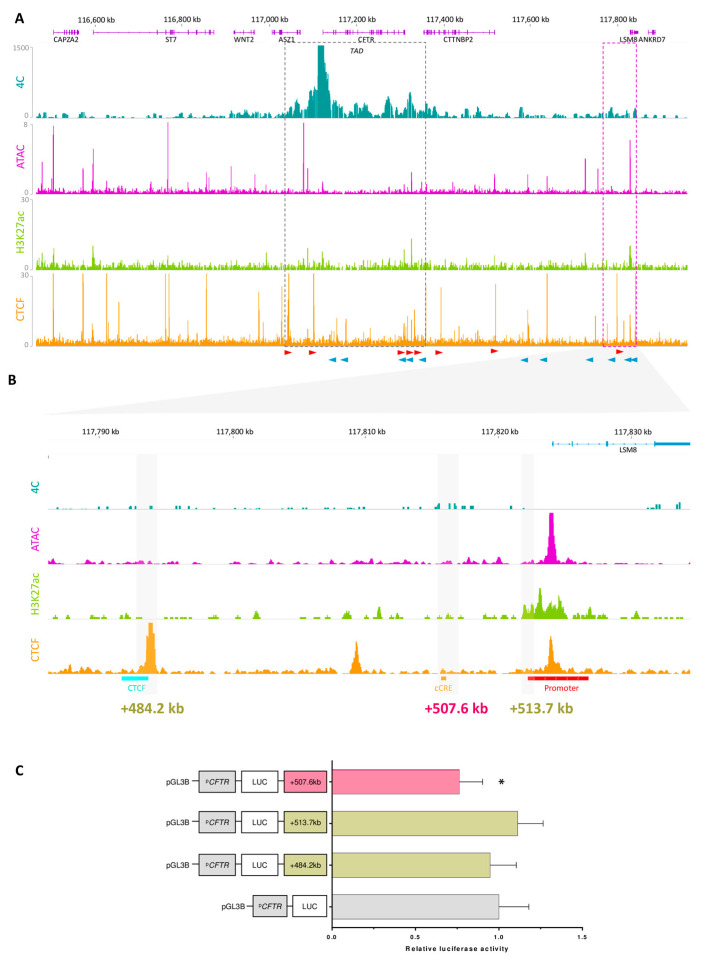
Exploration of regulatory elements beyond TAD boundaries. (**A**) Frequencies of *CFTR* promoter interactions were determined by 4C-seq in Capan-1 cells. ATAC-seq and CUT&RUN-seq were performed for the histone marks H3K27ac in Capan-1 cells and CTCF in Caco-2 cells. The orientation of each CTCF site is indicated by a blue or a red arrow. A region of interest has been selected in pink and zoomed in (**B**). The ENCODE cCREs database indicated three putative functional elements: a CTCF site in blue, an enhancer-like site in orange, and a promoter-like site in red. (**C**) These three regions were selected for use in luciferase assays. Capan-1 cells were transfected with luciferase reporter constructs containing the *CFTR* basal promoter (PCFTR; 787 bp) and cCREs. We observed a silencer effect in the +507.6 kb region. The luciferase data are shown relative to the *CFTR* basal promoter vector (set to 1). The error bars represent the standard deviation (SD; *n* = 9), * <0.05 using unpaired *t*-tests. Data were aligned to the hg19 genome.

Although many CRE–promoter interactions occur within the same TAD, it has been shown that some CREs can interact with promoters in different TADs, which is called boundary stacking [32]. Taking this into account, we extended our analysis of chromatin data beyond the scope of the *CFTR* TAD (Figure 7A). Peak calling analysis of the 4C-seq data revealed a region of interest near the *LSM8* gene. The promoter of the *CFTR* gene significantly interacts with a region located at +485 kb from the last codon of the *CFTR* gene (Table S1). To gain insight into the functional role of this region, which was mapped to 4C, ATAC-seq, H3K27ac, and multiple CTCF peaks, the region was aligned to the ENCODE database (https://screen.encodeproject.org/, accessed on 27 October 2023) (Figure 7B).

Analysis of the 4C data revealed that the higher signal region mapped to a CTCF peak at +484.2 kb and to a peak at +507.6 kb. Mapping to the ENCODE cCREs database showed that it corresponds to a predicted CRE region. This region also encompasses the promoter of the *LSM8* gene, which has an active chromatin signal in our data. Using these elements, we selected these three regions for functional assay testing (Figure 7C). They were subsequently cloned and inserted into the pGL3-Basic vector with the minimal *CFTR* promoter, and a luciferase assay was then performed. As expected, the CTCF site had no effect on the observed activity. A similar observation was made for the +513.7 kb region corresponding to the *LSM8* promoter. However, for the +507.6 kb region, which corresponds to the predicted CRE region, a silencer effect was observed (−1.32-fold). Those preliminary results demonstrate that while most CRE–promoter interactions occur within the same TAD, interactions can also extend beyond TAD boundaries, emphasizing the importance of exploring inter-TAD interactions for a comprehensive understanding of gene regulation.

### 2.6. Cell Type Specificity

**Figure 8:**
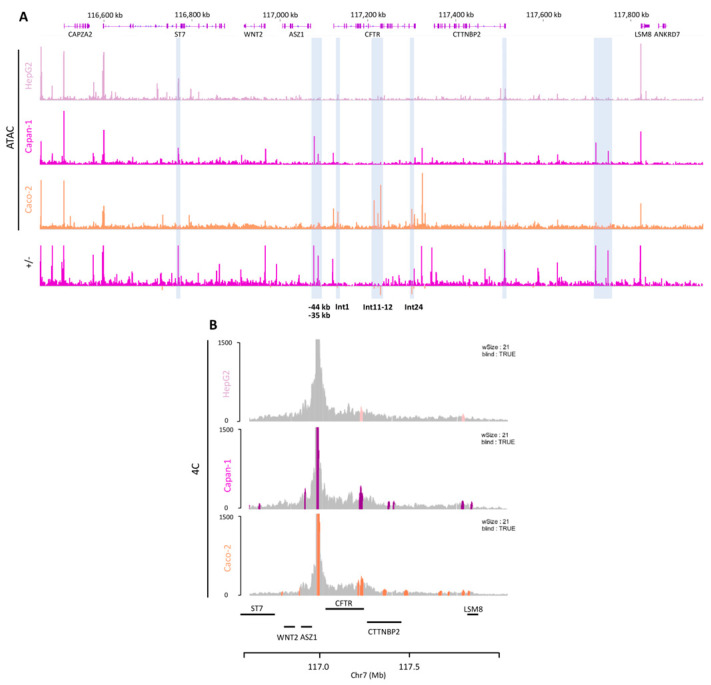
Tissue specificity of *cis*-regulation. (**A**) ATAC-seq data for Capan-1, Caco-2, and HepG2 cells to compare the tissue specificity of OCRs. HepG2 cells do not express the *CFTR* gene, and no OCR is detected at the *CFTR* locus. BigWig comparison (+/−) of Capan-1 and Caco-2 data revealed few differences (highlighted in blue), especially at the *CFTR* locus. −44 kb and −35 kb are specific for Capan-1 cells, and introns 1, 11, 12, and 24 are specific for Caco-2 cells. (**B**) *CFTR* promoter interaction frequencies were determined by 4C-seq in Capan-1, Caco-2, and HepG2 cells. HepG2 cells show only random interactions, whereas Capan-1 and Caco-2 cells show a specific profile. Significant regions are highlighted by colored peaks with shared and unique regions (alphaFDR; 0.4). Data were aligned to the hg19 genome.

To gain further insights into tissue-specific regulation of *CFTR* expression, we intended to compare data from other cell types. This allows us to identify tissue-specific characteristics. ATAC-seq data were generated for the intestinal Caco-2 cell line, and publicly available data for HepG2 cells were retrieved [33] (Figure 8A). Caco-2 cells are derived from a colorectal adenocarcinoma and express the *CFTR* gene, whereas HepG2 cells are derived from hepatocellular carcinoma and do not express the *CFTR* gene [34].

As expected, a comparison of open chromatin data profiles from *CFTR*-expressing cells and a cell line that does not express the gene revealed a notable difference in the profiles (Figure 8A). In HepG2 cells, no significant signal was observed around the *CFTR* locus. In the case of the *CFTR*-expressing cells, we observed a greater degree of similarity within the TAD, although interestingly, there were also some differences. A BigWig comparison analysis (Figure 8A, +/−) between Capan-1 and Caco-2 cells revealed some significant differences. The OCR signal in Caco-2 cells is more pronounced within intronic regions of the *CFTR* gene, confirming previous data that identified enhancers within introns 1, 11, 12, 24, and 26 [13,24,31,35]. For the Capan-1 cells, the majority of the peaks were observed outside the *CFTR* gene. Notably, there was a significant disparity between the two cell lines, with the −44 kb and *CFTR* 3′ regions lacking OCR peaks in Caco-2 cells. This finding appears to be specific to the pancreas. Differences were also observed in the OCR peaks outside the TAD. For example, the peaks located between the *CTTNBP2* and *LSM8* genes are exclusive to Capan-1 cells.

**Figure 9:**
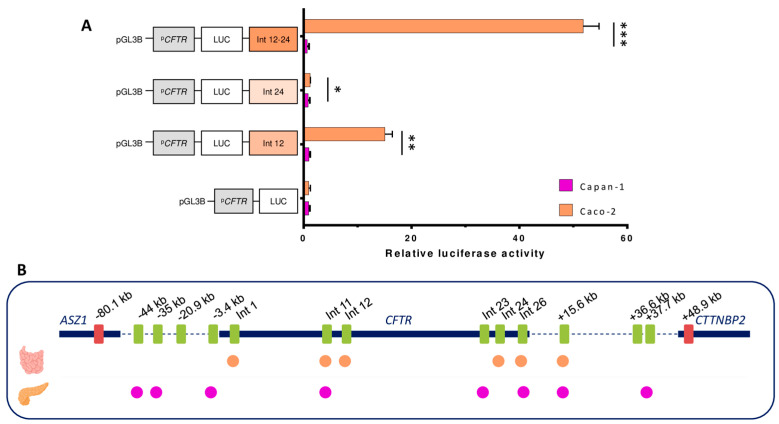
Activity of regulatory regions in Caco-2 and Capan-1 cells. (**A**) Capan-1 and Caco-2 cells were transfected with luciferase reporter constructs containing the CFTR basal promoter (PCFTR; 787 bp) and CREs. We observed an enhancing effect in Caco-2 cells for introns 12 and 24 and the combination, whereas no effect was detected in Capan-1 cells. The luciferase data are shown relative to the CFTR basal promoter vector (set to 1). The error bars represent the standard deviation (SD; *n* = 9), * <0.001; ** <1 × 10^−15^; *** <1 × 10^−19^ using unpaired *t*-tests. (**B**) Linear view of the CREs involved in CFTR regulation in the small intestine, with a higher proportion of intronic CREs. In the pancreatic cells, active CREs are present upstream from the CFTR gene as well as in the last introns of the gene and downstream.

The 4C-seq data from Caco-2 and HepG2 cells (Figure 8B) highlight a difference between cells expressing the *CFTR* gene and HepG2 cells, where there are more random interactions, and they decrease as we move away from the promoter. The profiles of Capan-1 and Caco-2 cells appear to be similar. PeakC analysis was conducted on both datasets, revealing identical interactions as well as some distinct interactions. For example, a significant peak encompassing intron 24, which is absent in Capan-1 cells, is observed in Caco-2 cells (Figure 8B). We show with ATAC-seq data that the major difference in the open chromatin regions between pancreatic and intestinal cells is in the cluster of introns 11 and 12 and within intron 24. A previous publication demonstrated that the OCR in intron 12 has a significant enhancing effect and that the combination of the OCRs in introns 12 and 24 has a notable effect on Caco-2 cells [24,31]. We repeated the luciferase reporter assay in both cell types (Figure 9A). In Caco-2 cells, intron 12 increased luciferase activity by 15-fold, and the combination of intron 12 and intron 24 increased luciferase activity by 50-fold. In contrast, no increase in luciferase activity was observed in Capan-1 cells in response to either of these regions. These enhancers hence appear specific to intestinal cells.

These chromatin analyses revealed that the *cis*-regulatory elements involved in the long-distance regulation of the *CFTR* gene exhibit a degree of tissue specificity, as illustrated in Figure 9B.

## 3. Discussion

To gain insight into the regulatory landscape of the *CFTR* gene, we set out to generate a 3D genome characterization in pancreatic cells. The *CFTR* gene has complex and incompletely described gene regulation at the temporal and tissue scales, and distal regulatory elements have been shown to regulate gene expression [14]. The best-described long-range regulatory mechanism involves the lung and intestine, but there is a considerable lack of long-range regulatory mechanisms in the pancreas, one of the first organs to display temporal CF-related symptoms early in life (even in utero).

Importantly, there is a lack of relevant pancreatic models that express the *CFTR* gene; for example, the widely used Panc1 cell line does not express enough of the *CFTR* gene [36]. Consequently, there is a lack of available chromatin data. To resolve this, we generated ATAC, H3K27ac, and 4C data in Capan-1 cells, a cell line of which we demonstrate *CFTR* gene expression.

For informative genome conformation studies, it is crucial to integrate multiple, complementary assays. The ATAC assay provides information on accessible DNA regions; however, these data must be contextualized with histone mark mappings to determine the functional role of the region. 4C provides insight into chromatin interactions. However, it is important to consider that not all interactions necessarily lead to transcriptional activation [37]. By combining these approaches, we identified potential regulatory regions. It remains, however, important to investigate whether all the active regions surrounding the *CFTR* gene are truly involved in its regulation. This step, which is crucial for determining the target gene, represents a significant challenge in understanding long-range regulation.

To address this issue, we decided to use the ABC predictive model [16]. For several years, numerous computational methods for determining gene–enhancer links have been developed and are still being developed. The ABC model is widely used because of the ease of use made by developers, and it has also been validated by users [38,39,40,41]. Nonetheless, the ABC model uses ATAC, H3K27ac, and Hi-C data and thus does not identify other CREs as silencers or epromoters, which are promoters with an enhancer function [42]. The predictive model helps us to prioritize the candidate regions. Four distal enhancers are predicted to regulate the *CFTR* gene; indeed, in this model, each gene is regulated by an average of 2.8 enhancers [17].

The 4C data provide complementary information on the 3D interactions of the *CFTR* promoter to validate our candidate regulatory regions, as recommended in a previous publication [3]. The +15.6 kb region is a known DHS previously described in pulmonary and intestinal cells as an enhancer-blocking region [20]. Figure S2 shows that in Caco-2 cells, enhancer blocking limits the propagation of H3K27ac. In Capan-1 cells, we confirmed the presence of an OCR, which is marked by H3K27ac and interacts with the *CFTR* promoter. By definition, an enhancer blocking is located between an enhancer and the protected promoter. In the case of the +15.6 kb region, we observed a small ATAC and H3K27ac just downstream, representing the +37.7 kb region, which could be an enhancer blocked by the +15.6 kb region. In the native context, in the presence of the +15.6 kb region, the +37.7 kb region does not have an enhancer effect and slightly reduces the activity in the luciferase assay.

The −44 kb and −35 kb regions predicted to target the *CFTR* gene correspond to both H3K27ac and ATAC peaks, but no major interaction was observed in 4C data. The −44 kb region shows an enhancer effect, but not the −35 kb region, which has a similar effect to that of the +37.7 kb region. From previous studies, we know that there is also an enhancer-blocking upstream of the promoter at −20.9 kb, which corresponds to a 4C peak in Capan-1 cells but not an ATAC peak [20,43]. It remains to be determined whether this cCRE is active in pancreatic cells, as the CTCF data are from Caco-2 cells. The last cCRE identified by chromatin data is located in intron 26 and has not been identified by the ABC model, but it corresponds to the 4C, ATAC, and H3K27ac peaks and has increased activity by 1.8-fold in the luciferase assay.

Because *cis*-regulation is hosted by a chromatin module, we wanted to add information about TFs to obtain a complete picture of this landscape [28]. We aligned TFs from JASPAR, as there is no ChIP-seq data for whole-genome exocrine pancreas-specific TFs in Capan-1 cells. It would be of interest to perform ChIP with HNF1B, Ptf1a, and FOXA1/2, as these proteins are more common in the active CRE. The STRING database (https://string-db.org/, accessed on 13 September 2024) revealed that HNF1B and Ptf1a are associated with one another (Figure S3). On the basis of the chromatin module, we also provide information on the cooperative effect of the CRE [25]. Indeed, the combination of CREs has a synergistic effect. The activity of each individual enhancer is lower than that of the combination [27]. Using Cas9-based genome engineering efficiently delivered through VLPs, we were able to confirm the role of the −44kb region in an endogenous setting. Although we demonstrate an endogenous impact of the individual deletion of the −44 kb region, considering the importance of the chromatin module, it would be interesting to go further by individually deleting the other CREs, as well as performing multiple deletions to determine whether the impact on *CFTR* expression is more pronounced than what we observe here. Subsequently, chromatin states will need to be assessed to understand the consequences of CRE loss. It will be important to define their individual roles, as they may play a role in bridging elements, as tethering elements can, or act as facilitators in multi-enhancer clusters [44,45]. Indeed, it has been shown that elements can lack intrinsic activity and still contribute to gene expression [46,47].

The majority of CRE–gene interactions are located within 100–500 kb of the TSS [16,48]. Hence, we wanted to extend our characterization beyond the TAD. Interestingly, we identified a significant long-range interaction 500 kb downstream of the TSS. By observing the orientation of the CTCF sites and based on the processed loop extrusion, we noticed that this region lies within the neighboring chromatin loop [49]. Studies have demonstrated the possibility of cross-TAD interactions, notably through boundary stacking [32]. Therefore, characterizing cCREs outside the TAD would be interesting. In the luciferase reporter assay, we also measured the silencer effect of this +507.6 kb region, which was predicted to be functional by the ENCODE database. In the database, it was annotated as an enhancer, but it is important to note that there is no silencer category. They are less well characterized than enhancers, and we must explore the binding of repressive TFs while considering that the luciferase assay performed is far from the native context. Additionally, it will be necessary to explore the region using technologies, such as those developed by Brosh et al., that account for larger DNA sequences, including both the target gene and its regulatory elements [50].

Importantly, *cis*-regulation is a tissue-specific process, and the 3D chromatin architecture changes depending on the cell type. In HepG2 cells, OCR and 4C signals are absent, whereas in Caco-2 and Capan-1 cells, chromatin architecture is important for regulating *CFTR* gene expression. Notably, only 19% of enhancer–gene pairs are shared across distinct cell types [17]. Our observations are consistent with this hypothesis, as the CREs identified in Caco-2 cells are not present in Capan-1 cells. It is important to note that our data were obtained from Capan-1 cell lines. Further studies are needed using more relevant models to strengthen our findings. Primary cells would be ideal, although they are much more challenging to work with and engineer [51].

**Figure 10:**
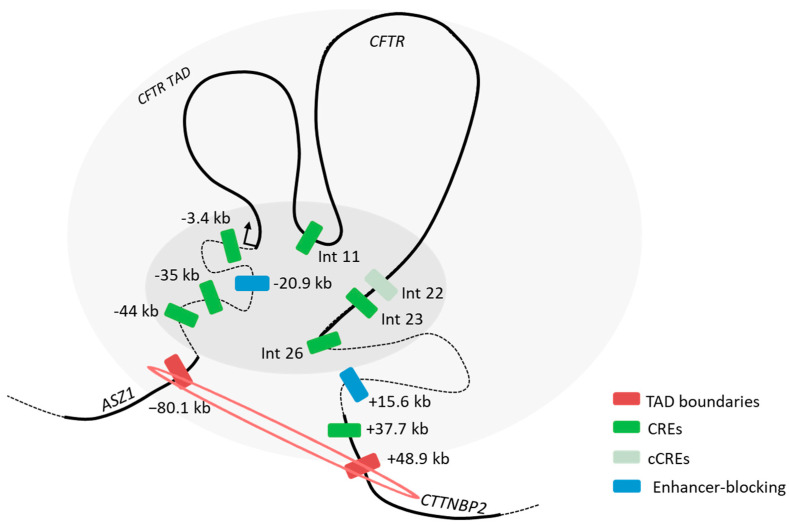
Schematic overview of the *CFTR* regulatory landscape. We provide a predictive three-dimensional model of *CFTR* regulation in pancreatic cells. Chromatin loops are represented with black lines, with coding sequences as thick lines. TAD boundaries are highlighted in red, candidate CREs in light green, and CREs in green. Enhancer-blocking elements are shown in blue.

In summary, we have provided chromatin genome-wide data in pancreatic Capan-1 cells, and our focus on the *CFTR* locus provides novel insights on the distal regulation of the *CFTR* gene with the identification of several CREs, as illustrated in Figure 10. In light of the increasing interest in non-coding variations in complex genetic diseases, a deeper understanding of chromatin architecture is crucial for advancing our knowledge in this field.

## 4. Materials and Methods

### 4.1. Cell Culture

The following three cell lines were used: Capan-1, which is derived from pancreatic adenocarcinoma; Caco-2, which is derived from colorectal adenocarcinoma; and HepG2, which is derived from hepatocellular carcinoma. Cells were grown in Dulbecco’s modified Eagle medium supplemented with 10% fetal bovine serum. The samples were incubated at 37 °C with 5% CO_2_. The cells are frequently tested for mycoplasma contamination.

### 4.2. ATAC-Seq

Omni-ATAC-seq libraries were generated as previously described [52]. A total of 5.10^4^ pelleted cells were resuspended in cold lysis buffer and incubated on ice for 3 min. Transposition was performed with buffer containing tagment DNA enzyme (Illumina, San Diego, CA, USA) at 37 °C and 1000 rpm for 30 min. DNA was purified via a MinElute PCR purification kit (Qiagen, Hilden, Germany). Libraries were amplified via NEBNext High-Fidelity 2 × PCR Master Mix (New England Biolabs, Ipswich, MA, USA) and Nextera adapters (Illumina). The PCR products were purified via a QIAquick PCR purification kit (Qiagen). Libraries were quantified via Qubit (Thermo Fisher, Waltham, MA, USA), and fragment sizes were analyzed via a bioanalyzer (Agilent, Santa Clara, CA, USA). Libraries were paired-end sequenced via Illumina sequencing (NextSeq500, Illumina). The generated paired FastQ reads were analyzed via the atacseq pipeline version 0.12 with default parameters and mapped to the hg19 reference genome (https://github.com/iwc-workflows/atacseq, accessed on 10 March 2024). After filtering and duplicate removal, peaks are called via macs2. A BigWig file contains the coverage file. For HepG2 cells, we downloaded ATAC-seq fastQ from [33] (GSE 139190).

### 4.3. CUT&RUN-Seq

CUT&RUN-seq libraries were generated as previously described [53]. A total of 5 × 10^5^ cells were incubated with activated concanavalin A beads (Bangs Laboratories, Fishers, IN, USA) for 10 min at 4 °C. The cells were resuspended in buffer containing 0.06% digitonin (Calbiochem, Darmstadt, Germany), and specific antibodies against H3K27ac (Ab4729), CTCF (Active Motif 61311), and H3K27me3 (Millipore 07-449) were added and incubated at 4 °C for 2 h. pAG-MNase (1 ng/µL, Cell Signaling Technology, Danvers, MA, USA) was added, and the mixture was incubated for 1 h on ice. Unbound enzymes were washed, and calcium chloride (2 mm) was used to activate pAG-MNase for 30 min on ice. After incubation, stop buffer was added, and the samples were incubated at 37 °C for 30 min to release DNA fragments. Purification was performed via DNA purification buffers and spin columns (Cell Signaling Technology). End-repair and A-tailing were then performed via the KAPA HyperPrep Kit (Roche, Bâle, Switzerland). Next, 10 µL of 4X ERA buffer were added to the libraries, which were subsequently incubated at 20 °C for 30 min and at 50 °C for 60 min. Ligation of KAPA UDI adapters (0.3 µM) was performed at 20 °C for 1 h. Size selection was performed via AMPure XP beads (Beckman Coulter, Brea, CA USA) with double purification via the addition of HXP buffer (20% PEG 8000, 2.5 M NaCl). Amplified libraries are then obtained by adding HotStar ReadyMix 2X and primer mix 10X and performing 12 cycles of 98 °C for 15 min, 60 °C for 10 s, and 72 °C for 1 min. The PCR products were further purified via AMPure XP beads. Libraries were quantified via Qubit (Thermo Fisher), and fragment sizes were analyzed via a bioanalyzer (Agilent). Libraries were paired-end sequenced via Illumina sequencing (NextSeq500, Illumina). The generated paired FastQ reads were analyzed with the cutandrun pipeline version 0.8 with default parameters and mapped to the hg19 reference genome (https://github.com/iwc-workflows/cutandrun, accessed on 10 March 2024). After filtering and duplicate removal, peaks are called via macs2. A BigWig file contains the coverage file.

### 4.4. 4C-Seq

4C-seq libraries were generated as previously described [22]. A total of 7.5 × 10^6^ cells were cross-linked with 2% formaldehyde (37%) for 10 min. Chromatin is first digested with Csp6I (200 U, Thermo Fisher) restriction enzyme and then with DpnII (200 U, New England Biolabs). Ligation was performed with 100 U of T4 DNA Ligase (Promega, Madison, WI, USA) at 16 °C. DNA purification was performed via NucleoMag clean-up and size selection beads (Macherey-Nagel, Düren, Germany). Inverse PCRs were performed via the Expand™ Long Template PCR System (Roche) with the primers listed in Table S2. The PCR products were quantified with a Qubit instrument (Thermo Fisher), and the fragment size was analyzed with a BioAnalyzer (Agilent). Libraries were sequenced at 75 bp single-end via Illumina sequencing (MiniSeq Illumina). The generated FastQ data were analyzed with the Pipe4C version 1.1.4 pipeline [22]. Data were aligned to the hg19 reference genome. A wig file containing the normalized data is available for visualization via genome browsers. Peak calling was performed with R script peakC (version 0.2) [54].

### 4.5. ABC Model

The activity-by-contact (ABC) prediction model is used to predict CRE promoter links [16]. ABC requires an open chromatin file and an active chromatin region file. BAM files free of duplicates from ATAC-seq analysis were used to generate a fragment file, which was then converted to a tagAlign file to match the required input format. The ABC pipeline was run with default parameters using the hg19 reference genome as the input. The data used were ATAC-seq and H3K27ac data from Capan-1 cells, with the power law used as the contact metric. The ABC score is calculated via the following equation:

$$\frac{Activity\;of\;E\times Contact\;frequency\;E-P}{Sum\;of\;(Activity\times Contact\;Frequency)\;over\;all\;candidate\;elements\;within\;5\;Mb}$$

The resulting CRE–promoter prediction links were filtered to obtain only those involving the CFTR promoter, and a paired BED file containing the predictions was produced.

### 4.6. Plasmid Construction

Regions of interest were amplified via CloneAmp HiFi PCR Premix and then inserted via In-Fusion cloning (TaKaRa, Kusatsu, Japan) into the pGL3-Basic Vector (Promega). The CFTR promoter (787 bp) is cloned and inserted into the HindIII restriction site, which is upstream of the firefly luciferase cDNA (luc). The candidate regulatory regions are subsequently cloned and inserted into the BamHI or XhoI restriction site. The PCR primers used are listed in Table S3.

CRISPR (clustered regularly interspaced short palindromic repeats) guides were designed using CRISPick and selected based on efficacy as well as predicted fidelity. Spacer sequences including compatible overhangs for cloning were ordered as single-strand DNA oligos (IDT, see Table S4) and annealed in buffer B (Thermo Fisher). Annealed oligos were phosphorylated using T4 polynucleotide kinase (Thermo Fisher) and ligated into a BpiI-restricted sgRNA expression backbone (pBluescriptSKII-U6-sgRNA F+E scaffold, Addgene #74707).

### 4.7. Reporter Gene Assay

A total of 2.5 µg of plasmid (4:1 ratio, plasmid of interest: pCMV-Bgal, internal control) was reverse transfected into 2.8 × 10^5^ cells with Lipofectamine 3000 using 2 µL of p3000 in 12-well plates. Each condition was performed in triplicate, and three independent transfections per construct were conducted. Seventy-two hours after transfection, the cells were lysed with 1X passive lysis buffer (Promega). The cell lysates were clarified, and 20 µL of protein extract was used for the luciferase assay and 50 µL for the beta-galactosidase colorimetric assay. Luciferase assay reagents were purchased from Promega, and Varioskan (Thermo Fisher) was used as a plate reader. Relative luciferase activity was calculated, and Student’s *t*-test was performed.

### 4.8. CRISPR/Cas9 Deletion

MLV-based virus-like particles (VLPs) were ordered from Leuven Viral Vector Core and produced as previously described [55]. In short, 7.106 HEK293T cells were seeded in five 10 cm diameter Petri dishes (BD Biosciences, Franklin Lakes, NJ, USA) and quadruple transfected with sgRNA/epegRNA/ngRNA expression plasmids, MLV-gag-pol, VSV-g, and gag-cargo fusion expression constructs following the ratios described by Mangeot et al., typically withholding the BaEVRless envelope and supplementing with additional VSV-G expression plasmid using PEI (PEI, Polysciences Europe, Warrington, PA, USA) [56]. The GagMLV-Cas9 plasmid used was a kind gift from David Liu (pCMV-MMLVgag-3xNES-Cas9; Addgene #181752) [57]. Supernatant containing lentiviral particles was harvested 48 h and 72 h post transfection, filtered through a 0.45 µm pore-size filter, and concentrated using centrifugation in Vivaspin columns at 3000× *g*. VLP productions were stored at −80 °C until further use.

Moreover, 10,000 Capan-1 cells were incubated with the VLPs for 10 min and then cultured under normal cell culture conditions. After 24 h, the medium was refreshed, and the cells were grown until sufficient numbers were obtained to perform DNA and RNA extractions. Homozygous deletion was confirmed by PCR followed by Sanger sequencing. Primers are listed in Table S4. CFTR gene expression was assessed by RT-qPCR using ONEGreen FAST qPCR Premix (Table S4).

## Data Availability

Sequencing data from this study are available at NCBI GEO under accession numbers GSE284199, GSE284200, and GSE284414.

## Supplementary Materials

The following supporting information can be downloaded at: https://www.mdpi.com/article/10.3390/ijms26083788/s1. Reference [58] is cited in the Supplementary Materials.

## Abbreviations

The following abbreviations are used in this manuscript:

4C	circular chromosome conformation capture
ABC	activity-by-contact
ATAC	assay for transposase-accessible chromatin
CBAVD	congenital bilateral absence of vas deferens
CF	cystic fibrosis
CFTR	cystic fibrosis transmembrane conductance regulator
CFTR-RD	CFTR-related diseases
cCRE	candidate *cis*-regulatory elements
CRE	*cis*-regulatory elements
CRISPR	clustered regularly interspaced short palindromic repeats
CTCF	CCCTC-binding factor
DHS	DNase I hypersensitive site
CUT&RUN	cleavage under targets and release using nuclease
gRNA	guide RNA
OCR	open chromatin region
PTM	post-translational modifications
TAD	topologically associating domain
TF	transcription factor
TSS	transcription start site

## References

[1] Abascal F., Acosta R., Addleman N.J., Adrian J., Afzal V., Aken B., Ai R., Akiyama J.A., Jammal O.A., The ENCODE Project Consortium (2020). Perspectives on ENCODE. Nature.

[2] Moore J.E., Purcaro M.J., Pratt H.E., Epstein C.B., Shoresh N., Adrian J., Kawli T., Davis C.A., Dobin A., Kaul R. (2020). Expanded encyclopaedias of DNA elements in the human and mouse genomes. Nature.

[3] Gasperini M., Tome J.M., Shendure J. (2020). Towards a comprehensive catalogue of validated and target-linked human enhancers. Nat. Rev. Genet..

[4] Lopez Soriano V., Dueñas Rey A., Mukherjee R., Coppieters F., Bauwens M., Willaert A., De Baere E., Genomics England Research Consortium (2024). Multi-omics analysis in human retina uncovers ultraconserved *cis*-regulatory elements at rare eye disease loci. Nat. Commun..

[5] Zaugg J.B., Sahlén P., Andersson R., Alberich-Jorda M., de Laat W., Deplancke B., Ferrer J., Mandrup S., Natoli G., Plewczynski D. (2022). Current challenges in understanding the role of enhancers in disease. Nat. Struct. Mol. Biol..

[6] The 100,000 Genomes Project Pilot Investigators (2021). 100,000 Genomes Pilot on Rare-Disease Diagnosis in Health Care—Preliminary Report. N. Engl. J. Med..

[7] Pang B., van Weerd J.H., Hamoen F.L., Snyder M.P. (2023). Identification of non-coding silencer elements and their regulation of gene expression. Nat. Rev. Mol. Cell Biol..

[8] El-Seedy A., Ladeveze V. (2024). CFTR complex alleles and phenotypic variability in cystic fibrosis disease. Cell Mol. Biol..

[9] Sermet-Gaudelus I., Girodon E., Vermeulen F., Solomon G.M., Melotti P., Graeber S.Y., Bronsveld I., Rowe S.M., Wilschanski M., Tümmler B. (2022). ECFS standards of care on CFTR-related disorders: Diagnostic criteria of CFTR dysfunction. J. Cyst. Fibros..

[10] Simmonds N.J., Southern K.W., De Wachter E., De Boeck K., Bodewes F., Mainz J.G., Middleton P.G., Schwarz C., Vloeberghs V., Wilschanski M. (2024). ECFS standards of care on CFTR-related disorders: Identification and care of the disorders. J. Cyst. Fibros..

[11] Gosalia N., Neems D., Kerschner J.L., Kosak S.T., Harris A. (2014). Architectural proteins CTCF and cohesin have distinct roles in modulating the higher order structure and expression of the CFTR locus. Nucleic Acids Res..

[12] Smith E.M., Lajoie B.R., Jain G., Dekker J. (2016). Invariant TAD Boundaries Constrain Cell-Type-Specific Looping Interactions between Promoters and Distal Elements around the CFTR Locus. Am. J. Hum. Genet..

[13] Smith A.N., Barth M.L., McDowell T.L., Moulin D.S., Nuthall H.N., Hollingsworth M.A., Harris A. (1996). A regulatory element in intron 1 of the cystic fibrosis transmembrane conductance regulator gene. J. Biol. Chem..

[14] Blotas C., Férec C., Moisan S. (2023). Tissue-Specific Regulation of CFTR Gene Expression. Int. J. Mol. Sci..

[15] Becq F., Fanjul M., Merten M., Figarella C., Hollande E., Gola M. (1993). Possible regulation of CFTR-chloride channels by membrane-bound phosphatases in pancreatic duct cells. FEBS Lett..

[16] Fulco C.P., Nasser J., Jones T.R., Munson G., Bergman D.T., Subramanian V., Grossman S.R., Anyoha R., Doughty B.R., Patwardhan T.A. (2019). Activity-by-contact model of enhancer–promoter regulation from thousands of CRISPR perturbations. Nat. Genet..

[17] Nasser J., Bergman D.T., Fulco C.P., Guckelberger P., Doughty B.R., Patwardhan T.A., Jones T.R., Nguyen T.H., Ulirsch J.C., Lekschas F. (2021). Genome-wide enhancer maps link risk variants to disease genes. Nature.

[18] Moisan S., Berlivet S., Ka C., Le Gac G., Dostie J., Férec C. (2016). Analysis of long-range interactions in primary human cells identifies cooperative CFTR regulatory elements. Nucleic Acids Res..

[19] Nuthall H.N., Moulin D.S., Huxley C., Harris A. (1999). Analysis of DNase-I-hypersensitive sites at the 3’ end of the cystic fibrosis transmembrane conductance regulator gene (CFTR). Biochem. J..

[20] Blackledge N.P., Carter E.J., Evans J.R., Lawson V., Rowntree R.K., Harris A. (2007). CTCF mediates insulator function at the CFTR locus. Biochem. J..

[21] Simonis M., Klous P., Splinter E., Moshkin Y., Willemsen R., de Wit E., van Steensel B., de Laat W. (2006). Nuclear organization of active and inactive chromatin domains uncovered by chromosome conformation capture–on-chip (4C). Nat. Genet..

[22] Krijger P.H.L., Geeven G., Bianchi V., Hilvering C.R.E., de Laat W. (2020). 4C-seq from beginning to end: A detailed protocol for sample preparation and data analysis. Methods.

[23] Rauluseviciute I., Riudavets-Puig R., Blanc-Mathieu R., Castro-Mondragon J.A., Ferenc K., Kumar V., Lemma R.B., Lucas J., Chèneby J., Baranasic D. (2024). JASPAR 2024: 20th anniversary of the open-access database of transcription factor binding profiles. Nucleic Acids Res..

[24] Ott C., Blackledge N.P., Kerschner J.L., Leir S.-H., Crawford G.E., Cotton C.U., Harris A. (2009). Intronic enhancers coordinate epithelial-specific looping of the active CFTR locus. Proc. Natl. Acad. Sci. USA.

[25] Martinez-Ara M., Comoglio F., Steensel B.V. (2024). Large-scale analysis of the integration of enhancer-enhancer signals by promoters. eLife.

[26] Osterwalder M., Barozzi I., Tissières V., Fukuda-Yuzawa Y., Mannion B.J., Afzal S.Y., Lee E.A., Zhu Y., Plajzer-Frick I., Pickle C.S. (2018). Enhancer redundancy provides phenotypic robustness in mammalian development. Nature.

[27] Kim S., Wysocka J. (2023). Deciphering the multi-scale, quantitative *cis*-regulatory code. Mol. Cell.

[28] van Mierlo G., Pushkarev O., Kribelbauer J.F., Deplancke B. (2023). Chromatin modules and their implication in genomic organization and gene regulation. Trends Genet..

[29] McCarthy V.A., Ott C.J., Phylactides M., Harris A. (2009). Interaction of intestinal and pancreatic transcription factors in the regulation of CFTR gene expression. Biochim. Biophys. Acta (BBA) Gene Regul. Mech..

[30] Zhang Z., Ott C.J., Lewandowska M.A., Leir S.-H., Harris A. (2012). Molecular mechanisms controlling CFTR gene expression in the airway. J. Cell. Mol. Med..

[31] Collobert M., Bocher O., Le Nabec A., Génin E., Férec C., Moisan S. (2021). CFTR Cooperative *Cis*-Regulatory Elements in Intestinal Cells. Int. J. Mol. Sci..

[32] Hung T.-C., Kingsley D.M., Boettiger A.N. (2024). Boundary stacking interactions enable cross-TAD enhancer–promoter communication during limb development. Nat. Genet..

[33] Tak Y.E., Horng J.E., Perry N.T., Schultz H.T., Iyer S., Yao Q., Zou L.S., Aryee M.J., Pinello L., Joung J.K. (2021). Augmenting and directing long-range CRISPR-mediated activation in human cells. Nat. Methods.

[34] Gheldof N., Smith E.M., Tabuchi T.M., Koch C.M., Dunham I., Stamatoyannopoulos J.A., Dekker J. (2010). Cell-type-specific long-range looping interactions identify distant regulatory elements of the CFTR gene. Nucleic Acids Res..

[35] Rowntree R.K., Vassaux G., McDowell T.L., Howe S., McGuigan A., Phylactides M., Huxley C., Harris A. (2001). An element in intron 1 of the CFTR gene augments intestinal expression in vivo. Hum. Mol. Genet..

[36] Chambers J.A., Harris A. (1993). Expression of the cystic fibrosis gene and the major pancreatic mucin gene, MUC1, in human ductal epithelial cells. J. Cell Sci..

[37] Yang J.H., Hansen A.S. (2024). Enhancer selectivity in space and time: From enhancer–promoter interactions to promoter activation. Nat. Rev. Mol. Cell Biol..

[38] Mummey H.M., Elison W., Korgaonkar K., Elgamal R.M., Kudtarkar P., Griffin E., Benaglio P., Miller M., Jha A., Fox J.E.M. (2024). Single cell multiome profiling of pancreatic islets reveals physiological changes in cell type-specific regulation associated with diabetes risk. bioRxiv.

[39] Hecker D., Behjati Ardakani F., Karollus A., Gagneur J., Schulz M.H. (2023). The adapted Activity-By-Contact model for enhancer–gene assignment and its application to single-cell data. Bioinformatics.

[40] Hoellinger T., Mestre C., Aschard H., Le Goff W., Foissac S., Faraut T., Djebali S. (2023). Enhancer/gene relationships: Need for more reliable genome-wide reference sets. Front. Bioinform..

[41] Ying P., Chen C., Lu Z., Chen S., Zhang M., Cai Y., Zhang F., Huang J., Fan L., Ning C. (2023). Genome-wide enhancer-gene regulatory maps link causal variants to target genes underlying human cancer risk. Nat. Commun..

[42] Malfait J., Wan J., Spicuglia S. (2023). Epromoters are new players in the regulatory landscape with potential pleiotropic roles. BioEssays.

[43] Smith A.N., Wardle C.J.C., Harris A. (1995). Characterization of DNASE I hypersensitive sites in the 120kb 5’ to the CFTR gene. Biochem. Biophys. Res. Commun..

[44] Batut P.J., Bing X.Y., Sisco Z., Raimundo J., Levo M., Levine M.S. (2022). Genome organization controls transcriptional dynamics during development. Science.

[45] Blayney J.W., Francis H., Rampasekova A., Camellato B., Mitchell L., Stolper R., Cornell L., Babbs C., Boeke J.D., Higgs D.R. (2023). Super-enhancers include classical enhancers and facilitators to fully activate gene expression. Cell.

[46] Thomas H.F., Kotova E., Jayaram S., Pilz A., Romeike M., Lackner A., Penz T., Bock C., Leeb M., Halbritter F. (2021). Temporal dissection of an enhancer cluster reveals distinct temporal and functional contributions of individual elements. Mol. Cell.

[47] Thomas H.F., Feng S., Haslhofer F., Huber M., García Gallardo M., Loubiere V., Vanina D., Pitasi M., Stark A., Buecker C. (2025). Enhancer cooperativity can compensate for loss of activity over large genomic distances. Mol. Cell.

[48] Xie F., Armand E.J., Yao Z., Liu H., Bartlett A., Behrens M.M., Li Y.E., Lucero J.D., Luo C., Nery J.R. (2023). Robust enhancer-gene regulation identified by single-cell transcriptomes and epigenomes. Cell Genom..

[49] Merkenschlager M., Nora E.P. (2016). CTCF and Cohesin in Genome Folding and Transcriptional Gene Regulation. Annu. Rev. Genom. Hum. Genet..

[50] Brosh R., Laurent J.M., Ordoñez R., Huang E., Hogan M.S., Hitchcock A.M., Mitchell L.A., Pinglay S., Cadley J.A., Luther R.D. (2021). A versatile platform for locus-scale genome rewriting and verification. Proc. Natl. Acad. Sci. USA.

[51] Xu Y., Nipper M.H., Dominguez A.A., Ye Z., Akanuma N., Lopez K., Deng J.J., Arenas D., Sanchez A., Sharkey F.E. (2024). Reconstitution of human PDAC using primary cells reveals oncogenic transcriptomic features at tumor onset. Nat. Commun..

[52] Corces M.R., Trevino A.E., Hamilton E.G., Greenside P.G., Sinnott-Armstrong N.A., Vesuna S., Satpathy A.T., Rubin A.J., Montine K.S., Wu B. (2017). An improved ATAC-seq protocol reduces background and enables interrogation of frozen tissues. Nat. Methods.

[53] Skene P.J., Henikoff S. (2017). An efficient targeted nuclease strategy for high-resolution mapping of DNA binding sites. eLife.

[54] Geeven G., Teunissen H., de Laat W., de Wit E. (2018). peakC: A flexible, non-parametric peak calling package for 4C and Capture-C data. Nucleic Acids Res..

[55] Ibrahimi A., Velde G.V., Reumers V., Toelen J., Thiry I., Vandeputte C., Vets S., Deroose C., Bormans G., Baekelandt V. (2009). Highly Efficient Multicistronic Lentiviral Vectors with Peptide 2A Sequences. Hum. Gene Ther..

[56] Mangeot P.E., Risson V., Fusil F., Marnef A., Laurent E., Blin J., Mournetas V., Massouridès E., Sohier T.J.M., Corbin A. (2019). Genome editing in primary cells and in vivo using viral-derived Nanoblades loaded with Cas9-sgRNA ribonucleoproteins. Nat. Commun..

[57] Banskota S., Raguram A., Suh S., Du S.W., Davis J.R., Choi E.H., Wang X., Nielsen S.C., Newby G.A., Randolph P.B. (2022). Engineered virus-like particles for efficient in vivo delivery of therapeutic proteins. Cell.

[58] Sturgill D., Wang L., Arda H.E. (2024). PancrESS—A meta-analysis resource for understanding cell-type specific expression in the human pancreas. BMC Genom..

