# Supplementary material for: Cis-Regulation of the CFTR Gene in Pancreatic Cells
Source: Int J Mol Sci. 2025 Apr 17;26(8):3788. doi: 10.3390/ijms26083788 (PMC12027686; doi:10.3390/ijms26083788)
Supplement: Supplementary file 1 [file ijms-26-03788-s001.zip › ijms-3570691-supplementary.pdf]

Supplementary Materials Data

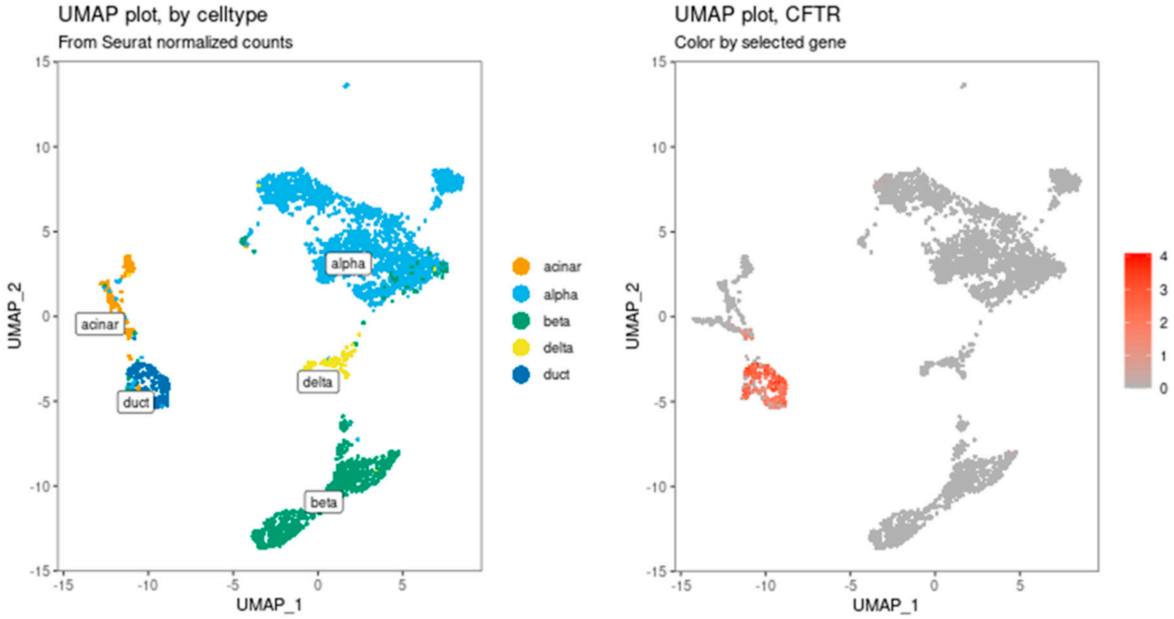

Figure S1. Single-cells CFTR expression. Single-cells CFTR expression. Single-cell RNA-seq data from the PancrESS dataset [1]. Uniform Manifold Approximation and Projection (UMAP) of transcript abundances. On the left: cells are color-coded by cell type, demonstrating clustering by cell type. On the right: cells expressing the gene of interest, CFTR, are highlighted in red, with the color intensity representing transcript abundance levels.

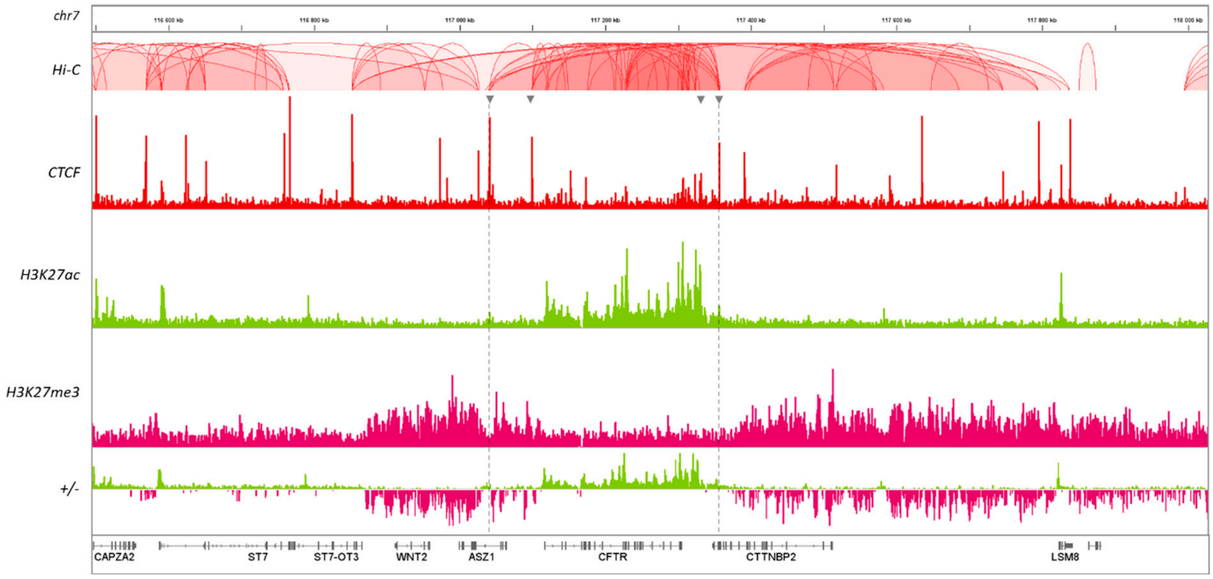

Figure S2. Chromatin data from Caco-2 cells. Hi-C data provide information of genome-wide chromatin interaction. CTCF immunoprecipitation allows to map CTCF binding. H3K27ac and H3K27me3 provide information of the chromatin state, acetylation is for active chromatin and trimethylation is for repress chromatin. TAD boundaries and enhancer blocking sites are indicated by grey arrow. We noticed the correlation of chromatin state and epigenetic marks.

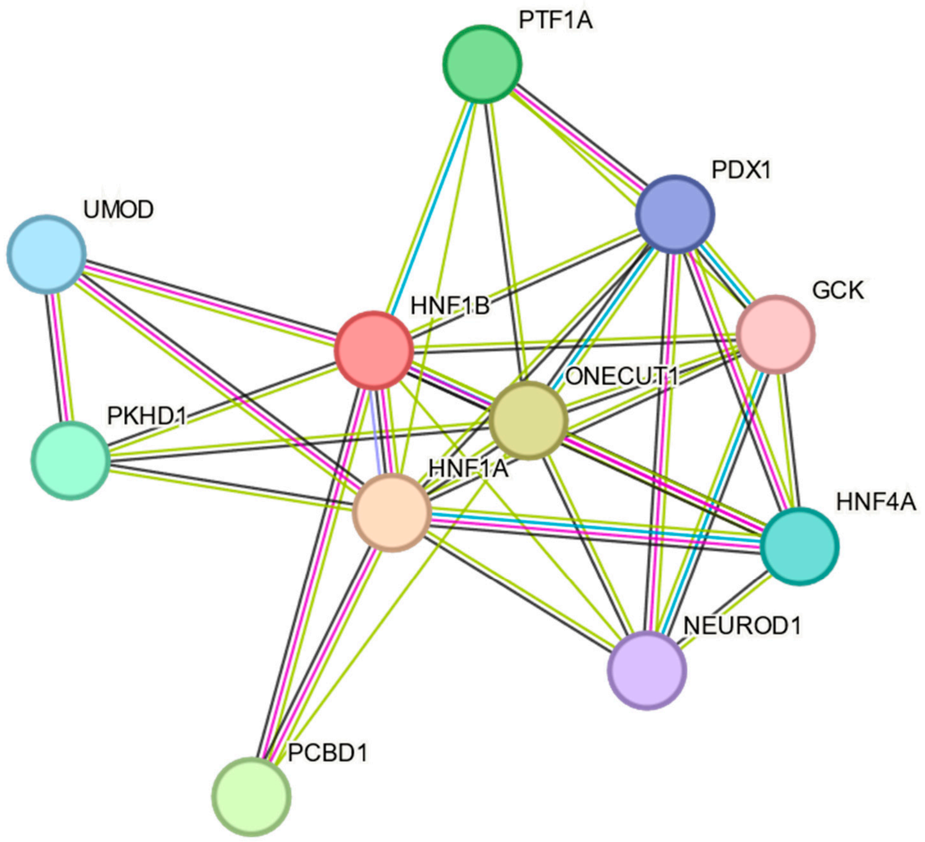

Figure S3. Transcription Factor Interaction Network. The STRING database provides an interaction network with HNF1b as the bait. The edges represent protein-protein associations.

Table S1. PeakC analysis of 4C data from Capan-1 cells with the CFTR promoter as bait (chr7:116,800,0006118,000,000, hg19).

	CRE	Genomic coordinates (hg19)
**1**		chr7:116843962-116853244
**2**	-80.1 kb	chr7:117042598-117048074
**3**		chr7:117057283-117064712
**4**	Promoter	chr7:117113481-117125179
**5**	+15.6 kb	chr7:117310255-117329893
**6**		chr7:117444432-117454749
**7**		chr7:117470041-117476691
**8**		chr7:117787647-117801774
**9**		chr7:117831712-117840409

Table S2. 4C-seq primers.

Primers Name	Primers Sequence
**4C_pCFTR_reading**	TACACGACGCTCTTCCGATCTGTCTTTCCATCATGAAATGCAC
**4C_pCFTR_nonreading**	ACTGGAGTTCAGACGTGTGCTCTTCCGATCTGAATTAAGCTCCAAAGAGGATC

Table S3. Primers for plasmid constructs.

CRE	Forward/reverse	Primers sequence
-44 KB	F	AAATCGATAAGGATCCGGTCATAGATGTGGAACATTCTG
	R	GATCAGATGTGGGATCCCCGCAAAATCTGCCATCATC
-35 KB	F	AAATCGATAAGGATCCCTCATGTAGCAGGCATTTGTG
	R	ATCGGTCGACGGATCCGTGTTTCCAATTTCTACCCAGAC
+15,6 KB	F	AAATCGATAAGGATCCCAGTGCCTGGCCAAGAATC
	F	ATCGGTCGACGGATCCGAGGTATCAGAAATCAGTGTGAG
+37,7 KB	R	AAATCGATAAGGATCCTACCACGTAAGTGAGATTATGTGG
	F	ATCGGTCGACGGATCCGAGTGGCTTCAAACCTTACTTATTC
-44 KB / -35 KB	R	CTAGCCCGGGCTCGAGGGTCATAGATGTGGAACATTCTG
	F	CAAATGCCTGCTACATGAGCCGCAAAATCTGCCATCATC
	F	GATGATGGCAGATTTTGCGGCTCATGTAGCAGGCATTTG
	R	GATCGCAGATCTCGAGCCAGAAGTTCACAAGACTG
+15,6 KB / +37,7 KB	F	AAATCGATAAGGATCCCAGTGCCTGGCCAAGAATC
	R	CATAATCTCACTTACGTGGTAGGAGGTATCAGAAATCAGTGTG
	F	CACACTGATTTCTGATACCTCCTACCACGTAAGTGAGATTATG
	R	AAATCGATAAGGATCCTACCACGTAAGTGAGATTATGTGG
-3,4 KB	F	AAATCGATAAGGATCCCTTTAGCACCCAAGCTCTTG
	R	ATCGGTCGACGGATCCCCTTATGGTTGTTGGTCACTTAC
Intron 11	F	AAATCGATAAGGATCCCTTAGTTCTCATGCTTTCTAGTGG
	R	ATCGGTCGACGGATCCGCAGTGGAGGATGGTTTGAAAG
Intron 12	F	AAATCGATAAGGATCCTGGAGAAGGTGGAATCACACTG
	R	ATCGGTCGACGGATCCGAAGACAGTATGCAAGAGCTACAT
Intron 18	F	AAATCGATAAGGATCCCTCCCCATTTCCCTCTCTCC
	R	ATCGGTCGACGGATCCGCTGGGCATTCGTCTTGGAG
Intron 24	F	AAATCGATAAGGATCCGCATTTCTCACTCTGGCTGG
	R	ATCGGTCGACGGATCCCCTGTCCAAACTAAGAAGACTTC
Intron 12-24	F	AAATCGATAAGGATCCTGGAGAAGGTGGAATCACACTG
	R	TACTGTCTTCGGATCCTCGACGCATTTCTCACTCTGGCTGG
	F	AAGGGCATCGTCGACCCTGTCCAAACTAAGAAGACTTC
	R	ATCGGTCGACGGATCCCCTGTCCAAACTAAGAAGACTTC
Intron 26	F	AAATCGATAAGGATCCATCTGAGCCATGTGGTGAGGTTGA
	R	ATCGGTCGACGGATCCTTCCTTTGAGCCTGTGCCAGTTTC
+484,2 KB	F	AAATCGATAAGGATCCGTACGAAGTATCAACTAGGAGAGC
	R	ATCGGTCGACGGATCCTCTTAGATAATTTAAAAAGCC
+507,6 KB	F	AAATCGATAAGGATCCGTGCCACAGAAAAAGATGGCC
	R	ATCGGTCGACGGATCCGAATAATGTAAGAAGGTATATAG
+513,7 KB	F	AAATCGATAAGGATCCGAGCATTACTTTGAGAGGTTTCGAC
	R	ATCGGTCGACGGATCCGTGTCTTCCGCTAATGAAAGAAC

Table S4. gRNA and PCR primers for CRISPR/cas9 deletion of -44 kb region.

**gRNA name**	**gRNA sequence**
**sgRNA-CRE1_F**	CAGAGAGGCCTGGGCTATCC
**sgRNA-CRE1_R**	GATATCAGACAACAAGTCTA
**PCR primers name**	**Primers sequence**
**Cis_CFTR_-44kb_Ext_F**	GGAAACAGGTAAGTTTTGTTCTG
**Cis_CFTR_-44kb_Ext_R**	GCTTTGTTCTCTGACCTAACC
**qPCR primers name**	**Primers sequence**
**CFTR_qPCR_F**	ATGCCCTTCGGCGATGTTTT
**CFTR_qPCR_R**	TGATTCTTCCCAGTAAGAGAGGC
**Actin_qPCR_F**	CTGGAACGGTGAAGGTGACA
**Actin_qPCR_R**	AAGGGACTTCCTGTAACAATGCA

Reference

1. Sturgill, D.; Wang, L.; Arda, H.E. PancrESS—A meta-analysis resource for understanding cell-type specific expression in the human pancreas. *BMC Genom.* **2024**, *25*, 76. https://doi.org/10.1186/s12864-024-09964-y.
